# The effect of sampling mode on response rate and bias in elite surveys

**DOI:** 10.1007/s11135-022-01406-9

**Published:** 2022-05-11

**Authors:** Matias López

**Affiliations:** Albert Hirschman Centre On Democracy, Graduate Institute of Geneva, Geneva, Switzerland

**Keywords:** Bias, Elite surveys, Non-probabilistic sampling, Random sampling, Response rates

## Abstract

The literature frequently recommends purposive sampling of elites based on the assumptions that random sampling negatively affects the response rate and that it induces bias. I test these assumptions drawing on metadata from 282 samples of political, economic, and social elites, and on microdata from 2,658 elites. First I use permutations to calculate confidence intervals for the expected response rate following each sampling method. Second, I estimate the effect of random sampling on the final response rate using a range of regression models. Finally, I compare the distributions of the estimators for the average age, the share of male elites, and elites’ ideology by simulating repeated random and purposive samples. Results indicate that both random and purposive sampling of elites generate sufficiently large samples, as well as consistent and unbiased estimators of population parameters. Contradicting methodological guidelines in the field, the conclusion is that random sampling of elites is efficient.

## Introduction

Elites are defined by their position in powerful organizations and their disproportional access to both economic and symbolic resources (Higley and Burton 2006; Higley and Pakulski 2012; López 2013; Reis and Moore 2005). Such an advantage alludes to the primacy of their thoughts and preferences over those of the general population (Amsden et al. 2012; Khan 2012; Reis and Moore 2005; Savage and Williams 2008). The preferences and opinions of elites do not tend to mirror those of average citizens (Gilens and Page 2014; Gulbrandsen 2018; Lupu and Warner 2020), and such deviation or low congruence is not constant nor easy to predict (Lupu and Warner 2021). Moreover, the preferences and subjective motivations of elites are relevant for a wide range of research questions in the social sciences, from democratization to policy making, conflict resolution, and beyond. All the latter make elite surveys a powerful source of data for social science research.

However, collecting data from quality samples of elites is hard because they constitute small, insulated, and heterogeneous populations. These traits make response rates more of a problem for surveys of elites if compared to surveys of the general population (Dillman et al. 2002; Groves 2006; Groves and Peytcheva 2008). Survey methodologists often associate response rates in the general population with the effect of survey mode (see Dillman et al. 2002 for a review), an issue that was addressed by Vis and Stolwijk (2020) regarding elite surveys as well.

More commonly, methodological discussions in elite research focus on how to approach elites (Bussell 2020; Dexter 2006; Gilding 2010; Goldstein 2002; Harvey 2010). The literature presents a generalized tendency to frame random sampling as too difficult or counterproductive for elite surveys (Bailer 2014; Cousin et al 2018; Harvey 2010; Hoffmann-Lange 2007, 2018; Mikecz 2012; Olivares et al. 2020; Rodríguez-Teruel and Daloz 2018; Walgrave and Joly 2018). Sometimes explicitly but more often implicitly, authors suggest that random sampling will fail to gather a sufficiently large sample of elites or generate biased samples.

A simple statistical argument for why random sampling would reduce the response rate of elites is that, assuming the hope of participation to be very low and a limited number of sampling attempts, the sampling distribution should converge towards a minuscule average sample. In what concerns sample validity, researchers commonly worry about the composition of their final samples being biased towards less powerful elites, or individuals that are less “representative” of the set of elites.

Following the above, purposive sampling^1^ is commonly regarded as more efficient in capturing more senior elites who effectively rule, whereas randomization could inflate samples with lateral actors in the elite world. In a nutshell, two assumptions against random sampling of elites prevail in the field: (i) random sampling of elites reduces the odds of collecting sufficiently large samples, and (ii) random sampling of elites are more likely biased. Despite their wide circulation, these and other methodological guidelines for elite surveys remain untested (Semenova 2018). The present study fills this gap by estimating whether, and to which extent, random sampling hurts studies of elites in terms of their response rate and sample composition.

Elites can be divided into subtypes, such as political elites, economic elites, and social or civil society elites, with further relevant subdivisions within them (Johansson and Uhlin 2020). The theoretical relevance of each group varies according to research agendas, but this distinction also renders practical impacts as some elites may be easier to sample than others.

In order to address the effect of sampling in elite studies I collected metadata and microdata from samples of elites in government, parties, business, and interest groups such as unions, churches and civil society organizations fielded between 1959 and 2020. The analysis of these data provides a clearer picture regarding the implications of probabilistic and non-probabilistic sampling strategies when conducting studies of elites.

The remainder of this article is as follows. I first review the main discussions regarding sampling methods for elite populations. I then present the data and methods utilized. The subsequent sections show results. I end the paper by discussing the implications of findings for future surveys of elites.

### Sampling elites

In 1959, James Robinson, a political science professor at Northwestern University, selected 100 offices of members of the Congress of the United States at random and sent three graduate students to Washington DC to interview them. Within one week, Robinson’s three person team had interviewed 45 members of Congress. By the end of the term, 90 out of the 100 randomly selected MPs had participated in his study (Robinson 1960). Having such a high quality randomized sample of the American Congress in such a short time may have gone from relatively easy to virtually impossible since then. The task of sampling elites increases in difficulty when accounting for more comprehensive definitions of elites, including powerful actors outside government branches.

Considering such a difficulty, Hoffmann-Lange (2007, 2018) famously formalized the positional method of elite identification in the following manner: First, the researcher should identify the most influential organizations within a country, then identify the positions of command within those, and finally list the individuals occupying such positions. Such a list can then be taken as a sample frame for an elite survey, i.e. an exhaustive list of traceable members of the population of interest. A recent derivation of the positional method of elite identification uses network analysis to estimate the boundaries of elite communities, as well as the core power nods within them (Larsen et al. 2017). These identification steps are key for assessing coverage, i.e. whether the potential participants indeed belong to the population of interest and whether members of such a population are not left unlisted.

After listing elites in a sample frame, researchers face the question of how to select potential participants. The randomization of participants makes the case for external validity, i.e. the account to which a sample statistic (ẋ) is informative of the value of a parameter (μ) describing the population from which the sample was drawn from. The central limit theorem (Laplace 1810) demonstrates how the values of ẋ calculated over randomized trials are normally distributed and centered at the true value of μ, or *E* (ẋ) = μ, making such an extrapolation feasible within established levels of uncertainty. When participants are selected using a criterion other than randomization, this property has to be assumed.

Notwithstanding, skepticism about randomization prevails amongst scholars of elites. For instance, Hoffmann-lange (2018:79) argues that random sampling is not an option because the “size and structure of elite populations are unknown.” This statement echoes a common confusion between methods of elite identification and sampling methods. Elite identification precedes sampling. It is the theory task of establishing set membership in a population of elites. Sampling methods provide criteria for selecting potential participants from the resulting pool of elites.

Researchers often imply that selecting elites at random is counterproductive because of their heterogeneity, small population size, reluctance to participate, amongst other obstacles (Cousin et al 2018; Tansey 2007; Rodríguez-Teruel and Daloz 2018). The potential effect of random sampling is even more pressing in comprehensive studies of elites in which powerful individuals in diverse groups are sampled. In these cases, researchers need to ensure that all subtypes of elites are well represented in their dataset. Furthermore, researchers worry about their final sample being imbalanced in favor of less powerful elites who may be more prone to participate in research but less “representative” of the population of elites.

In light of the former, when it comes to studying elites, researchers often opt for non-probabilistic sampling methods, i.e. those that do not rely on randomization. Rodríguez-Teruel and Daloz (2018) acknowledge that non-probabilistic sampling makes it harder for elite researchers to portray findings as externally valid. They nonetheless accept this limitation in aims of increasing the number of participants.

Different non-probabilistic sampling methods are available to researchers (see Daniel 2011). Either explicitly or implicitly, elite researchers tend to adopt purposive sampling, i.e. they target individuals on purpose in aims of reducing the aforementioned risks (Bakkalbasioglu 2020, Bussell 2020). Researchers may sample specific elite individuals on purpose because they believe that the data that they will provide will be informative of the traits of other elites and/or because they expect these individuals to participate, placing their criterion closer to convenience sampling (see Etikan et al. 2016 for implications of purposive vs convenience sampling). As an example, a sample of elites in the ruling party can be a dozen or so leaders understood to be influential, and a sample of economic elites can be another small set of powerful business leaders to which researchers built access to. Purposive sampling is often used in conjunction with some type of rank, in particular in the case of economic elites, privileging individuals at the top of the rank in the hope of getting estimates that are more representative of more powerful actors (e.g. Best et al. 2012). Respondent-driven sampling, a derivation of snow-balling, is yet another option that some researchers consider promising (Cousin et al. 2018).

Not uncommonly, researchers of elites that are more concerned with sample size contact all listed members of the elite population in an attempt to sample those who are available and willing to participate. Such a version of availability sampling is adopted in particular, but not exclusively, in surveys of parliamentary elites, precisely because these are small populations of typically a few hundred individuals or less (Bailer 2014).

Elite researchers commonly implement sampling quotas in order to model the sample after the distribution of elite groups in the population and/or to balance samples in the hope of reducing potential sources of bias (e.g. Stevens et al. 2006). The data gathered from non-random samples can also be balanced using propensity scores (see Ferri-García and Rueda 2018; Hansen and Bowers 2008) or simply by weighting elite groups in the data. However, the latter can be used to balance data even if randomization was used for the sampling of elites (examples in elite studies are Cao et al. 2019; López et al. 2020).

Above all, the preference for non-probabilistic sampling in studies of elites is grounded on the understanding of elites as a typical case of a hard to survey population (see Khoury 2020). Populations can be hard to survey for instance because they are too small, too dispersed, because demographic information about them is not easily available, because members are systematically uncooperative with researchers, or due to some combination of these and other unusual traits (Tourangeau et al. 2014). In order to access these populations, researchers have turned to innovative protocols, including sampling through social media (see Dosek 2021). While elites fit some aspects of hard to survey populations, the parallelism between them and more typical cases of such populations can be questioned, in particular because it is relatively easy to list and contact individuals holding formal positions of power. By comparing fieldwork notes from their studies of government officials and coca leaf growers in Chile and Bolivia, Alberti and Jenne (2019) highlight how intensive ethnographic immersion is often needed in order to be access more typical hard to survey populations while elites can be easily requested an interview through formal means (whether they will comply is yet another manner). Researchers have previously highlighted the relative simplicity of contacting elites and getting them to participate, in particular when populations are defined through their formal position (Dexter 2006; Groholt and Higley 1972). The super rich are harder to approach, but even in their case researchers have managed to create sample frames by using available records (Page et al. 2012). The option for purposive sampling is therefore generally not connected to problems of coverage of sample frames, but solely to expectations regarding elites’ participation.

It is reasonable to assume that purposive sampling would render large enough and balanced samples of elites. This does not imply that random sampling is not efficient. Random sampling should perform similarly to purposive sampling if, contrary to what many elite researchers believe, elites’ availability and willingness to participate are more or less normally distributed. If the latter is true, random sampling of elites should be as effective as purposive sampling, and therefore privileged due to the statistical properties it carries.

## Data and methods

The design aims at testing two hypotheses that mirror the expressed beliefs in the current literature:

### *H*_*1*_

Random sampling of elites severely compromises response rates.

### *H*_*2*_

Random sampling leads to sample bias.

The null hypotheses *H*_*0a*_ and *H*_*0b*_ state the opposite, that response rates following randomization are as high or higher than those following purposive sampling, and that randomization does not lead to sample bias. By sample bias I mean that the sampling method generates biased estimators of the characteristics of the populations. In order to test these hypotheses I use two datasets. The first is an original compilation of metadata from over 280 samples of elites. The second dataset accounts for the information of over 2,000 participants in surveys of party elites. In what follows I describe the data collection process for the original dataset in great detail. I then outline the statistical procedures utilized to test *H*_*1*_ and *H*_*2*_.

### Data collection

I compiled information from studies that administered standardized questionnaires in samples of elites. In addition to well-known studies of elites based on surveys, I searched “survey” + “elites” in Google Scholar in conjunction with sectors (e.g. “business,” “parliamentary”) and countries (e.g. “Russia,” “Canada”). Equivalent searches included terms such as “MP” for surveys of members of parliament and “CEO” for surveys of corporate elites. The data collection was restricted to samples of national elites,^2^ excluding samples of local elite populations (e.g. state legislators) and of international elites, such as those in the European Parliament or international civil servants. This decision privileges comparability across samples of elites that have similar positions at the top of the power structure in their countries. I also did not include samples of aspiring elites from surveys of political candidates. On several occasions, studies cited other elite surveys, which were then traced. The final dataset accounts for 282 samples of elites for which information about the response rate was available.

There are different ways of calculating response rates. However, the response rate for these relatively small samples simply reflect the share of sampled elites who participated in each study over all invited elites. Some studies report the average response rate between different samples of elites, either in more than one country or more than one elite population. This was the case of 83 samples. In these cases I inputted the average response rate of the full study in each sample.

I categorized surveys’ target populations in four groups: (1) “government elites,” which account for public officials in national executive bodies; (2) “party elites,” which cluster members of parliament and party leaders; (3) “economic elites,” which account for leaders in associations of business interest, top managers in the biggest companies within countries, and the super-rich; and (4) “social elites,” which cluster leaders in civil society, academia, churches, unions, and media. Samples of government and party elites make up for 64% of the data, followed by economic elites (19%) and social elites (17%).

In addition, for each sample I computed: (1) the type of sampling (random vs not-random), (2) the type of implementation (face to face, by mail, by phone, and mixed), (3) the N of invited individuals, (4) the extension of fieldwork in months, (5) the length of the questionnaire, and (6) whereas the sample was part of a multi-elite or comprehensive elite study (> 3 types of elites in total).

The adoption of randomization in elites’ selection does not imply that studies followed simple-random sampling. Most applied stratified random sampling. What is important for the present study is whether participants have a random probability of being selected or if they are selected on purpose. For this reason I coded all surveys relying mainly on some form of random sampling as belonging to the same treatment group. A description of these different characteristics of survey samples and how they are associated with response rates can be seen in Table 1.

**Table 1:** Average response rates according to survey mode

	Percentage using (%)	Average response rate (%)
Randomized sampling	40	80
Purposive/availability samping	60	50
Face to face administration	49	67
Mailed or online (self-administration)	27	50
Mixed administration (Face to face+phone)	24	39

**Fig. 1:**
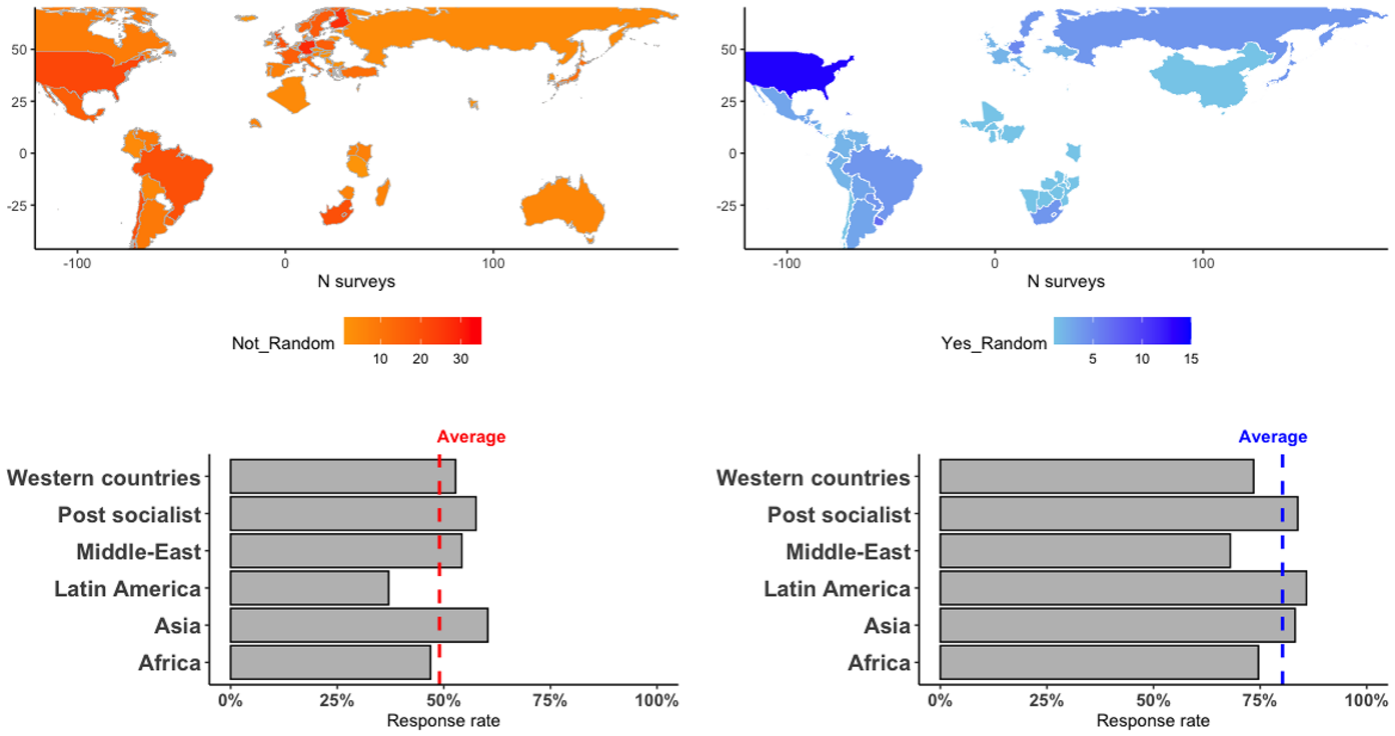
Geographic distribution of samples

The average duration of fieldwork was 115 days, the average questionnaire length was 172 questions, and the average sample size was 105. The dataset is organized on a country-year-sample basis, in which each row contains data from one sample of elites in one of the four groups. The geographic distribution of samples can be seen in Fig. 1.

All regions have cases of both random and purposive samples of elites, and several countries within them do as well. In order to account for differences between country-years I merged the data with information about the population size of countries in each year, their democratic status as coded by Boix (2003), and the level of income inequality as estimated by Solt (2020). As seen in Fig. 1, the average response rate is much greater in the group of random samples in the data. It is not reasonable to conclude from this that *H*_*1*_ is false, as several aspects of survey design could account for this difference. In what follows I outline the design for testing both *H*_*1*_ and *H*_*2*_.

### Testing *H*_1_

I test the hypothesis that randomization reduces the response rate of elite surveys using two procedures: a permutation test and regression models. I first conduct a permutation test in which I randomly select data from 10 samples of elites, half in each group (random and purposive sampling), and compute the baseline response rate in each subsample. In order to calculate a 95% confidence interval for the expected response rate in each group, I repeat this process 2,000 times, simulating how the two sampling methods perform in repeated applications. Confidence intervals portray the range of more plausible values for the average response rate in both groups. I use permutations to calculate a confidence interval for the p value over repeated one-way *T* tests. The benefit of using permutations, much in line with that of random sampling itself, is that the interpretation of results demands fewer and simpler assumptions.

In addition, I use regression models as an alternative method for estimating confidence intervals while controlling for different covariates. I model the metadata from elite surveys using OLS and multilevel regressions with both fixed and random slopes in a total of 21 models. Multi-level models cluster the data according to the country and decade in which the studies were conducted, therefore emphasizing comparison within countries and time. Seven core specifications were adapted to three slyly different model types: OLS, ML with fixed slopes, and ML with random slopes. The outcome variable is the response rate, ranging from 0 to 1. The goal of regression models is to explicitly account for the effect of covariates that are expected to influence the response rate, such as the type of elite population, implementation mode, duration of fieldwork, and country characteristics. The covariates included in the models are described in Table 2.

**Table 2:** Covariates in models

Additive section	N
*r* + *f*	279
*r* + *f* + *ss* + *pe* + *ee* + *ge* + *fw* + *me* + *d* + *p*	203
(r*f) + *ss* + *pe* + *ee* + *ge* + *fw* + *me* + *d* + *p*	203
*r* + *f* + *ss* + (r*pe) + (r*ee) + *ge* + *fw* + *me* + *d* + *p*	203
(r*f) + *ss* + (r*pe) + (r*ee) + (r*ge) + *fw* + *me* + *d* + *p*	203
*r* + *f* + *ss* + *pe* + *ee* + *ge* + *fw* + *me* + *d* + *p* + *q*	114
*r* + *f* + *ss* + *pe* + *ee* + *ge* + *fw* + *me* + *d* + *p* + *pop* + *gini* + *dem*	202

*r* Random sampling (0,1); *f* Face to face (0,1); *ss* Original sample size (including non-participants); *pe* Party elite (0,1); *ee* Economic elite (0,1); *ge* Government elite (0,1); *fw* Duration of field work in months; *me* Multi-elites in original study (0,1); *q* Questionnaire length (N questions); *d* Decade of the study as factor (1950…2020); p = sample from PELA (0,1); *pop* Population of the country in that year; *gini* Gini index in that year; *dem* Democratic status (0,1)

As noted in Table 2, I also control for whether the data comes from one of the PELA^3^ surveys (p) with Latin American parliamentary elites, as the project implemented random sampling and is prone to unusually high response rates^4^ (see Barragán et al. 2020).

### Testing *H*_2_

In order to test the assumption that random sampling generates biased samples of elites I use data from 2,658 participants in 39 samples of party elites. Data account for elites in 7 Latin American countries in which both random and purposive samples were implemented between the year 2000 and the year 2018. The data comes from five projects: PELA (see Barragán et al. 2020), NIED’s^5^ elite project (see López et al. 2020; Reis and Moore 2005), NUPRI-USP^6^’s project on elites (see Balbachevsky and Holzhacker 2011), the BLS^7^ (see Power and Zucco, 2012), and the “Encuesta Continua de Elites” project (Selios and Buquet forthcoming book). For a controlled comparison I only use the data from party elites, which was a common target population in the five projects. Within that population, the PELA, NIED, BLS, and Encuesta Continua de Elites datasets account exclusively for members of parliaments. Samples from the NUPRI-USP project account for members of parliament but also members of party executives.^8^ Descriptives can be seen in Table 3.

**Table 3:** Surveys and participants’ descriptives

	Treatment (Random sampling)	Control (Not random)
N participants	1,964	694
Countries (Year)	Argentina (04,08,13), Bolivia (03, 06,10), Brazil (05, 13), Chile (02,10, 14), Mexico (04,06, 10, 12, 15, 18), Uruguay (05, 13, 15), Venezuela (00)	Argentina (08), Bolivia (08), Brazil (08,13,17), Chile (08), Mexico (08), Uruguay (01, 02, 03, 04, 05, 06, 07, 09, 14, 17), Venezuela (08)
Projects	PELA, NIED	NUPRI-USP, BLS, Encuesta Continua de Elites

I address three individual characteristics asked by the surveys: age, sex, and self-reported ideology (standardized in a left–right 5-point scale^9^). In order to compare estimates from random and purposive sampling I simulate a population of 600 party elites in each country by selecting survey participants at random with replacement. Half of those come from surveys that implemented random sampling and the second half comes from surveys that implemented purposive sampling. Country membership is balanced in each simulated population. I then ran 1,000 samples of 100 elites from each population, each time computing the average age, proportion of males, and average ideology.

I assess bias by contrasting the mean estimation (ẋ) with proxies for the population parameters (μ) of average age and of the proportion of males. Bias is defined as  ẋ − μ, and I assume  ẋ − μ ≈ 0 as evidence of unbiasedness, considering that the values of μ are imputed by proxies. The proxy for age is the average age of members of parliament in the seven countries in the closest year to that of the survey when an exact match was not available. The proxy for the parameter of proportion of males is the share of male members of parliament in the Americas in 2010. Finally, I estimate confidence intervals for the parameters of the model *Ideology* = *α* + *β1.Male* + *β2.(Age 40–59)* + *β3.(Age 60 plus)* + *Ɛ* over repeated samples to test whether the different sampling methods affect the estimation of model parameters.

## Results

**Fig. 2:**
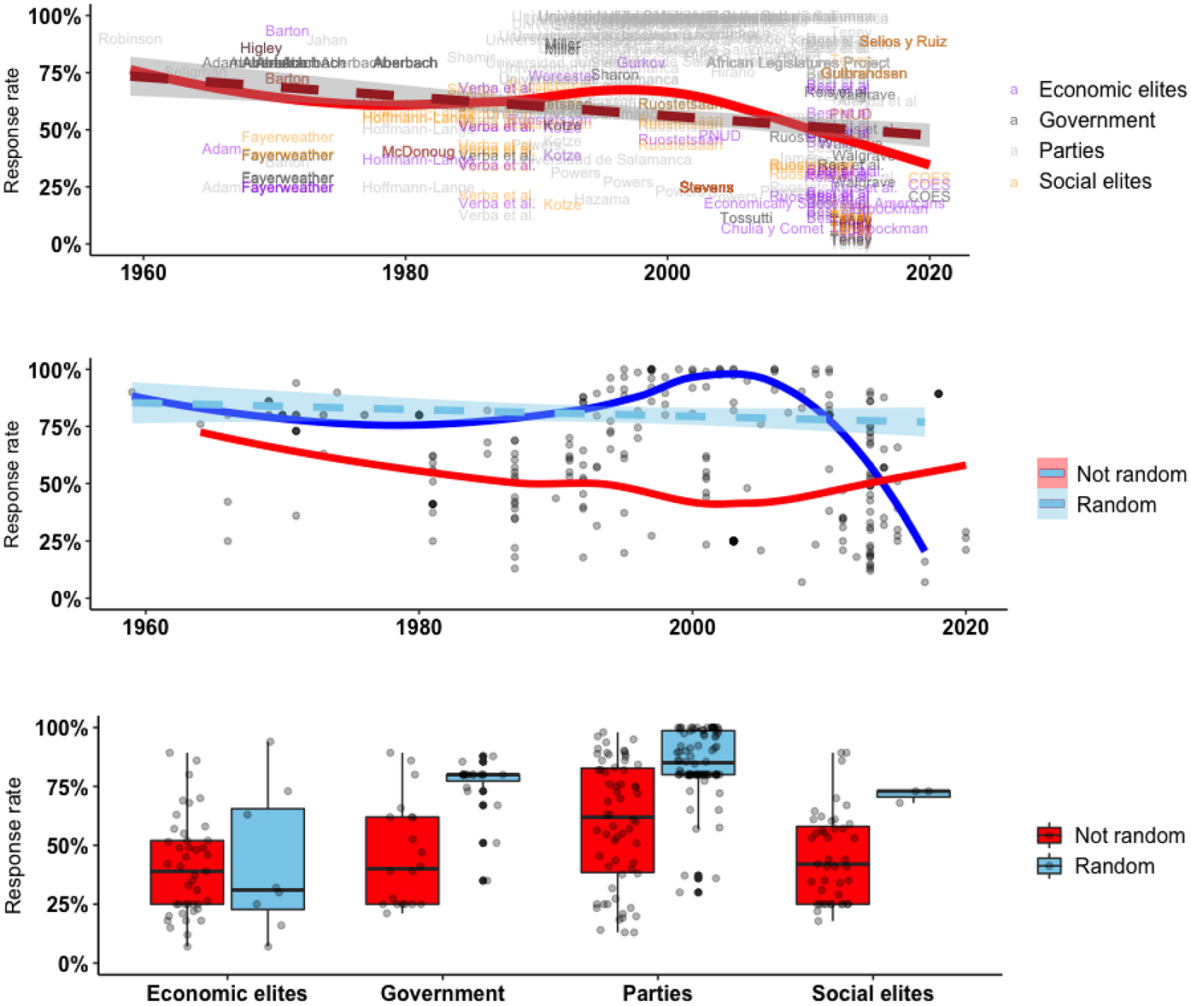
Response rates in time and per group

Figure 2 shows trends in the response rate of elites in time across sampling methods and across elite groups. While response rates among elites are clearly declining, the average remains above 50%, which is considered high even for surveys of the general population.

Labels in the upper plot in Fig. 2 flag the leading author or the project’s title for each observation. Repetitions are due to the same authors and projects having conducted multiple samples among different elite populations and years. Best et al. (2012), for instance, conducted samples of party and economic elites in multiple countries in a single year as a part of a large comparative project, while Powers (see Powers and Zucco 2012) conducted samples of Brazilian party elites in multiple years. The middle plot in Fig. 2 shows the same distribution with separate trend lines for each sampling method. The comparison portrays an observed advantage of random sampling until the 2010s. The boxplot shows how randomization is associated with higher response rates within the different elite groups as well, except among economic elites where the distribution is fairly similar.

**Fig. 3:**
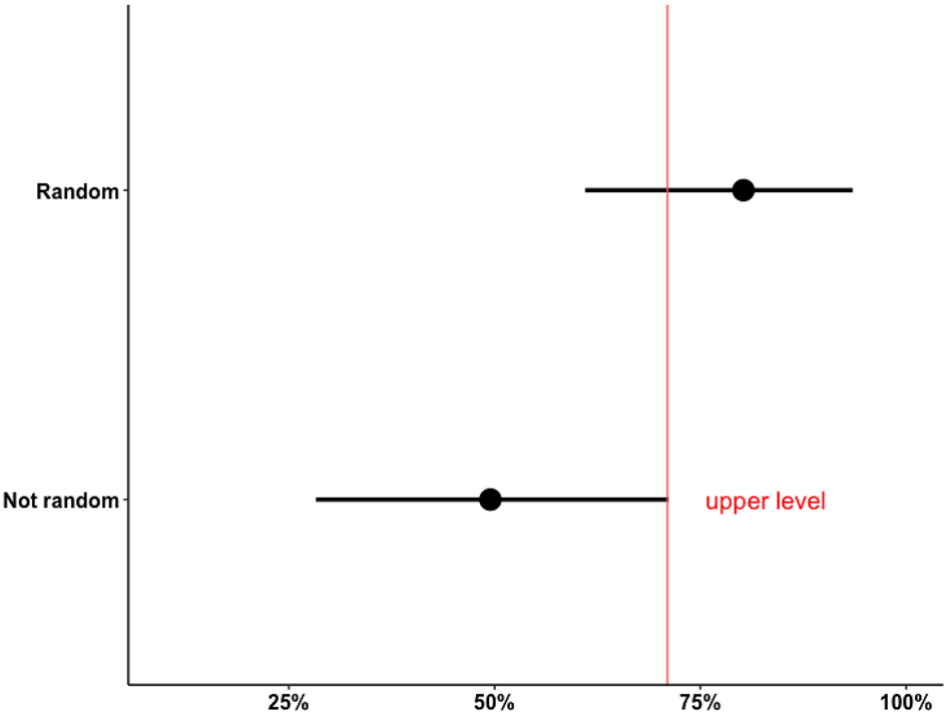
Average response rate in shuffled samples

The observed advantage of randomization seems to contradict previous publications on methods of elite sampling, which mostly portray it as impractical. As noted, this distribution does not constitute proof that random sampling outperforms purposive sampling, as there are other attributes of surveys’ design that should be accounted for. Figure 3 shows the confidence intervals for the expected response rate following both random sampling and other non-probabilistic alternatives, calculated through permutations.

**Fig. 4:**
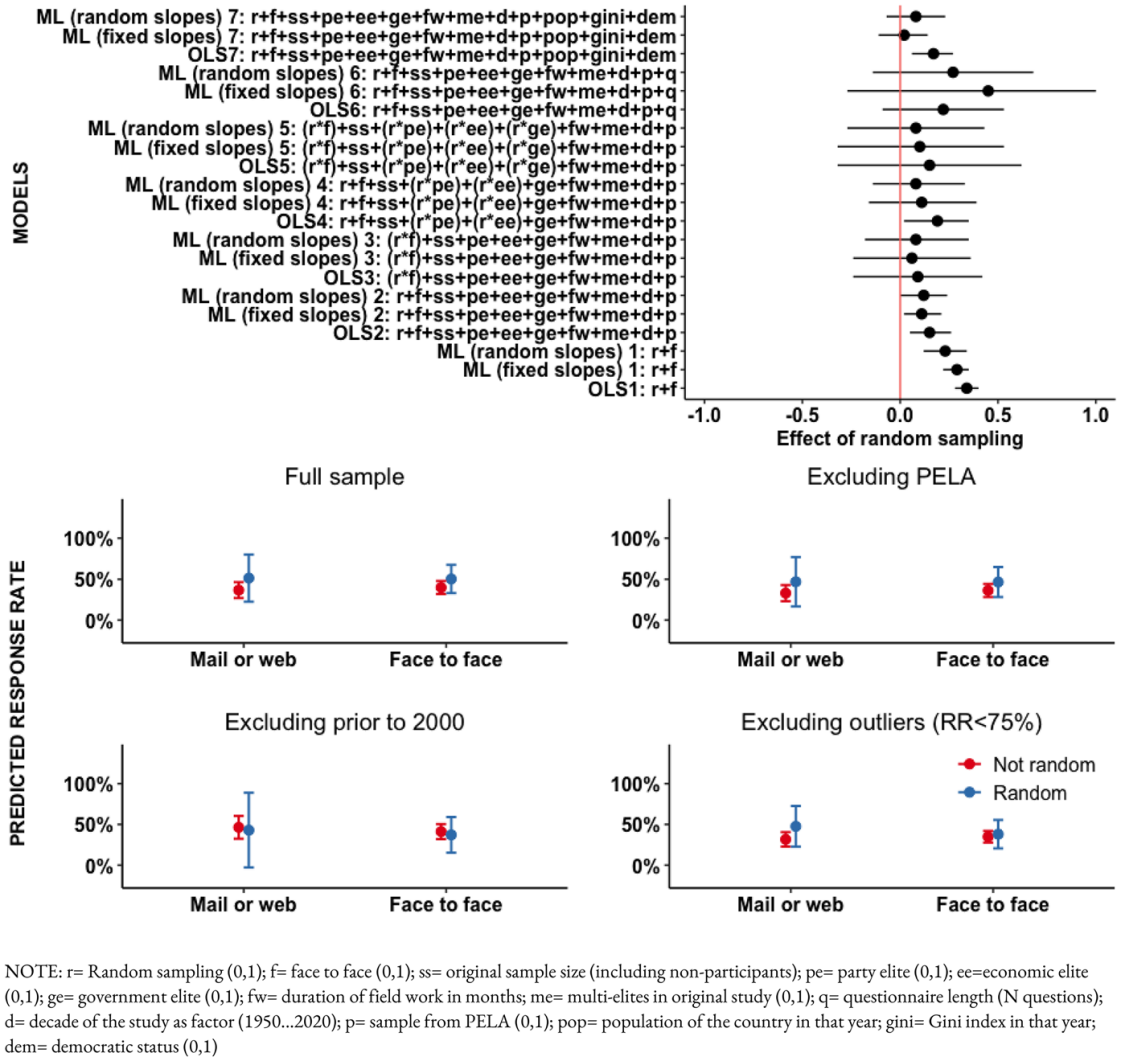
Regression estimates of the effect of random sampling

Confidence intervals calculated with permutations portray that the most extreme plausible average loss due to randomization is  − 10 percentage points in the response rate, assuming the confidence interval’s upper level in the control group and the lower level in the treatment group as the true expected response rates. The significance test indicates a probability of at least 99.9% of observing the present estimates if *H*_*0a*_ is true, i.e. if the expected response rate following random sampling is equal or higher than that expected following purposive sampling of elites. Figure 4 shows confidence intervals for the effect of random sampling and the predicted response rate for each group as estimated by regression models.

All models generate estimates that are highly expected in a distribution in which random sampling performs as well or better than purposive sampling in terms of the expected response rate. Models OLS 1, ML (fixed slopes) 1, ML (random slopes) 1 control only for face to face interviewing and continue to describe the observed advantage of random sampling. As controls are added to the model, estimates converge towards zero but are never reverted towards a disadvantage of random sampling, even when accounting for interaction terms in Models 3, 4, and 5. Models 6 portray wide confidence intervals due to less observations. Models 7 (on the top of the figure) include country characteristics and portray shorter confidence intervals and effects more clearly close or equal to zero.

Predicted response rates portray roughly equivalent results for surveys using different sampling strategies, in particular when considering face to face administration. There were only two^10^ randomized surveys using mailed-in questionnaires, which explains the large confidence intervals for that group. Models clearly overestimate the response rate in randomized mailed-in surveys of elites. The actual response rate of mailed-in elite surveys may also be inflated due to the lack of control regarding who actually filled the survey forms.

The predicted values for response rates using random sampling and purposive sampling overlap, suggesting that differences, if they exist, are not meaningful. Estimates are not sensitive to data from the PELA project, nor significantly affected by other particularly successful surveys with response rates above 75%, and also not driven by older surveys conducted prior to the year 2000. Overall, the evidence is highly expected under the null hypothesis.

### Effect on sample composition

**Fig. 5:**
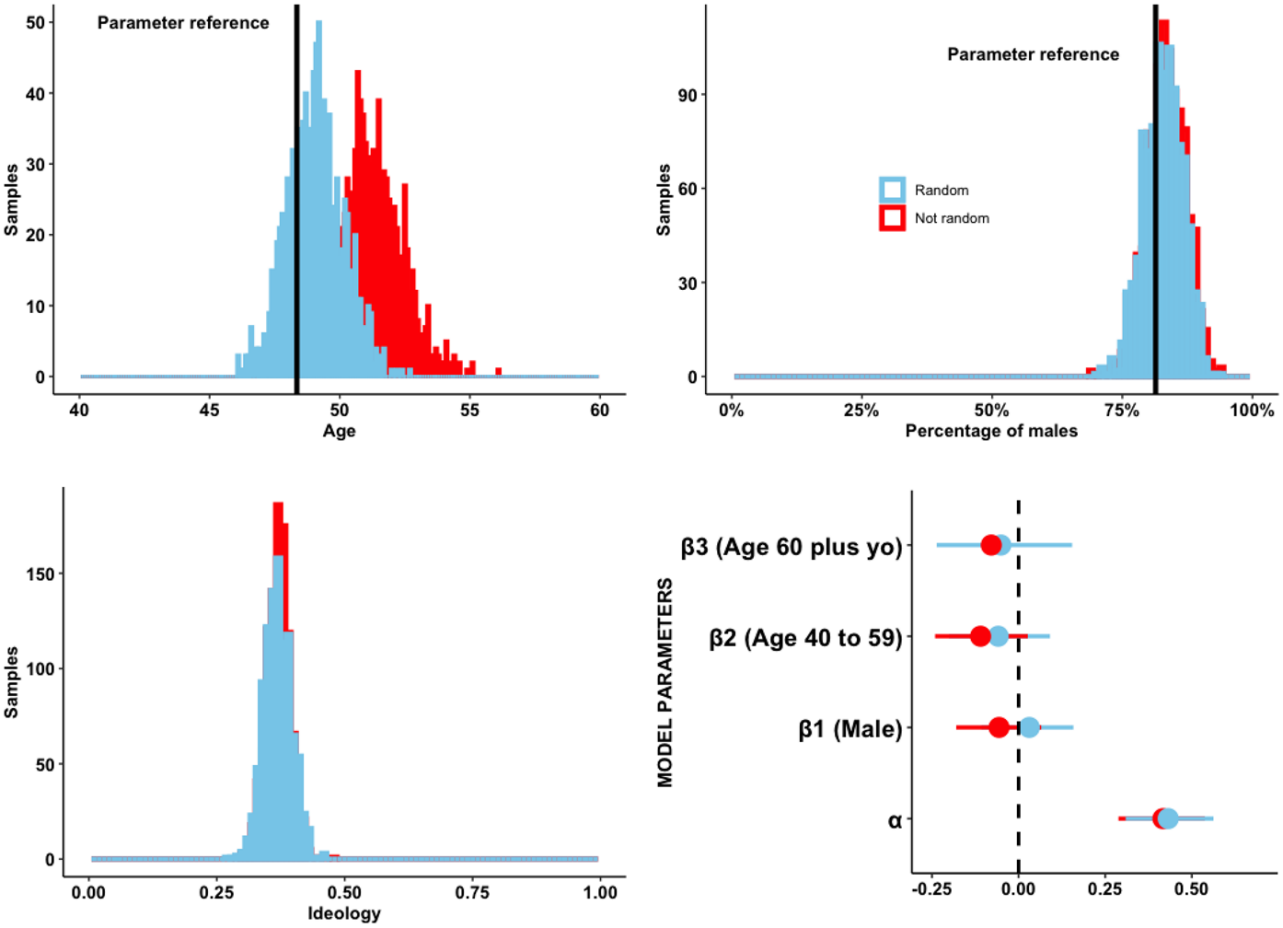
Distribution of estimates from simulations

Even if the average response rate does not differ substantially between random and purposive sampling, a second argument against random sampling of elites sustains that randomization may be a source of bias because the resulting sample composition will not resemble the population of elites due to skewness in willingness to participate and availability. Figure 5 shows the distribution of estimates of age, sex, and ideology accross samples of simulated elite populations which were originally selected at random and on purpose.

As shown in the upper-left part of Fig. 5, the estimator of the average age of elites in the random sampling group is centered at close proximity to the value of the parameter reference (ẋ− μ = 0.5), which indicates unbiasedness. The control group of non-random sampling generated a small bias of +2.5 years, i.e.older elites were slyly oversampled by purposive sampling. The estimator of elites’ average age in the treatment group of random sampling is also more consistent (*SD* = 1.08) if compared to the control group of purposive sampling (*SD* = 1.24). Confidence intervals in the treatment group cover the reference value for the population parameter of age 92% of the time, against 46% in the control group with data from purposive sampling.

The histogram in the upper-right section of Fig. 5 shows that both random and purposive sampling of elites produced unbiased and consistent estimators of the proportion of males in the population of elites. The lower-half part of Fig. 5 shows the distribution of the estimators for the average ideology of elites in a 0 to 1 left–right scale, as well as how the ideology of elites is predicted by participants’ age groups and sex across repeated samples. Results show that the estimators of elites’ ideology generated by random and purposive sampling converge in their distribution. Although the estimates of age in the control group are biased, the average regression coefficients for the effect of age groups on ideology converge among the two sampling methods. The estimated parameters for the effect of sex and the intercept also converge.

## Discussion and conclusion

Researchers commonly anticipate that elites will not be successfully captured by random sampling (Bailer 2014; Cousin et al. 2018; Rodríguez-Teruel and Daloz 2018). Such a prediction often follows two assumptions. The first is that random sampling of elites will compromise the final number of participants. The second is that randomization generates bias. Following the above, purposive sampling is commonly portrayed as a more effective method for collecting a sufficiently large and valid sample of elites (Hoffmann-Lange 2007, 2018; Walgrave and Joly 2018). The present study finds such assumptions to be inaccurate.

Results indicate that purposive sampling is unlikely to outperform random sampling significantly. Confidence intervals calculated over repeated simulations portray that the negative effect of random sampling on the average response rate of elites is either nonexistent or very small. A variety of predictive models portrayed estimates that are highly expected under the null hypothesis, reinforcing the conclusion that random sampling does not cause a reduction in the final number of elite participants.

Concomitantly, simulations suggest that random sampling of elites produces consistent and unbiased estimators, which is not always true for purposive sampling. However, in general both sampling methods produced similar sample compositions, as well as estimators that converge both in their distribution and in their capacity to estimate the parameters of a model predicting elites’ ideology. In a nutshell, if a researcher were to estimate the association between elites’ ideology, age, and sex, she would most likely reach similar results independently of her option for random or purposive sampling.

The conclusions of the present study are therefore two-fold. On the one hand, findings indicate that researchers can confidently implement random sampling of elites if they also account for other tailoring guidelines that incentivize elites’ cooperation, such as face to face implementation (see Vis and Stolwijk 2020). This is consequential for the field of elite research because random sampling reduces the number of assumptions needed to interpret sample statistics in light of central tendencies. In other words, researchers can be more confident of the external validity of their findings when randomizing participants. On the other hand, results portrayed converging estimates from probabilistic and non-probabilistic samples of elites, although within a limited range of tested covariates.

Beyond statistics, some practical matters speak in favor of purposive sampling. For instance, randomization may demand more time and resources than other sampling methods because it distributes selection odds evenly among elites who may be geographically dispersed or in effect more insulated. Researchers may also opt for purposive sampling to target decision makers within a very specific policy or socialization context in order to estimate the chain of events that led to a particular outcome or the network of social relations centered in particular positions (Tansey 2007).

One counterintuitive finding was that, regardless of the sampling method adopted, elite surveys actually tend to have high response rates. Considering the latter, should we maintain our understanding of elites as hard to survey populations? There are important nuances in answering the latter. Elites can generally be considered hard to survey in the sense that they demand more from researchers if compared to the average individual. Elite researchers accomplish high response rates in part because they allocate much time and resources in getting each selected individual to comply with their study. They do so through multiple contacts, scheduling flexibility, and long periods of fieldwork (Mikecz 2012). Moreover, results show a significant downward tendency in the response rate of elite surveys, which indicates that this population is becoming even harder to survey. However, elites are not particularly hard to sample when compared with other recluse groups for which random sampling might indeed be unworkable.

The task of elite identification is time consuming and itself demanding of formal methods (Hoffmann-Lange 2007, 2018). Notwithstanding, the methods of elite identification should not be confused with sampling methods, as listing the members of a population is a prior step to that of selecting potential participants. All things considered, once the members of an elite population are identified, both random sampling and purposive sampling are feasible and efficient selection methods. However, random sampling should be privileged for its simplicity and statistical properties.

## Footnotes

1 Rodriguez-Teruel and Daloz (2018: 101), state that “elite survey must follow modes of non-probability sampling such as purposive sampling.” Mikecz (2012: 486), argues that “Given the very limited number and the influential position of these individuals, random sampling was [is] inappropriate.” Cousin et al. (2018: 237) argue that “those interested in doing survey research within elite populations face important challenges … especially if they aim for a controlled sampling procedure.”.

2 Two exceptions were geographically delimited surveys that aimed at capturing individuals who could be accounted as representative of national elites: a survey with affluent members of the Communist Party in Beijing (Chen 1999) and a survey of millionaires in a Chicago suburb (Page et al. 2013).

3 PELA stands for Parliamentary Elites of Latin America.

4 I found no explanation for PELA’s high response rates in the project’s methodological material, which can be accessed on https://oir.org.es/pela/en/. Access on November 17, 2021.

5 NIED stands for Interdisciplinary Network for the study of Inequality.

6 NUPRI-USP stands for Núcleo de Pesquisa em Relações Internacionais da Universidade de São Paulo.

7 BLS stands for Brazilian Legislative Survey.

8 These are not specified in the data and therefore cannot be removed.

9 The original scales in the surveys vary from 5 to 10 points. I harmonized the data in quintiles for comparison.

10 Both samples were conducted by Brookman at Stanford and his colleagues (2019), targeting economic elites in the United States. They received questionnaires back from 16% of the sampled business elites and 7% of super-rich individuals.
